# Correction to: Recombinant human interleukin-7 reverses T cell exhaustion ex vivo in critically ill COVID-19 patients

**DOI:** 10.1186/s13613-022-01007-7

**Published:** 2022-04-01

**Authors:** Frank Bidar, Sarah Hamada, Morgane Gossez, Remy Coudereau, Jonathan Lopez, Marie-Angelique Cazalis, Claire Tardiveau, Karen Brengel-Pesce, Marine Mommert, Marielle Buisson, Filippo Conti, Thomas Rimmelé, Anne-Claire Lukaszewicz, Laurent Argaud, Martin Cour, Guillaume Monneret, Fabienne Venet, RICO Study Group, Remi Pescarmona, Lorna Garnier, Christine Lombard, Magali Perret, Marine Villard, Sébastien Viel, Valérie Cheynet, Elisabeth Cerrato, Estelle Peronnet, Jean-François Llitjos, Laetitia Itah, Inesse Boussaha, Françoise Poitevin-Later, Christophe Malcus, Marine Godignon, Florent Wallet, Marie-Charlotte Delignette, Frederic Dailler, Marie Simon, Auguste Dargent, Pierre-Jean Bertrand, Neven Stevic, Marion Provent, Laurie Bignet, Valérie Cerro, Jean-Christophe Richard, Laurent Bitker, Mehdi Mezidi, Loredana Baboi

**Affiliations:** 1 Joint Research Unit HCL-bioMérieux, EA 7426 “Pathophysiology of Injury-Induced Immunosuppression”, Université Claude Bernard Lyon, 1-Hospices Civils de Lyon-bioMérieux, 69003 Lyon, France; 2 Anesthesia and Critical Care Medicine Department, Edouard Herriot Hospital, Hospices Civils de Lyon, 69437 Lyon, France; 3 Immunology Laboratory, Hôpital E. Herriot-Hospices Civils de Lyon, 5 place d’Arsonval, 69437 Lyon Cedex 03, France; 4 Centre International de Recherche en Infectiologie (CIRI), Inserm U1111, CNRS, UMR5308, Ecole Normale Supérieure de Lyon, Université Claude, Bernard-Lyon 1, Lyon, France; 5 Biochemistry and Molecular Biology Laboratory, Lyon-Sud University Hospital-Hospices Civils de Lyon, Chemin du Grand Revoyet, Pierre-Benite, France; 6 Centre d’Investigation Clinique de Lyon (CIC 1407 Inserm), Hospices Civils de Lyon, 69677 Lyon, France; 7 Medical Intensive Care Department, Hospices Civils de Lyon, Edouard Herriot Hospital, 69437 Lyon, France

## Correction to: Ann Intensive Care (2022) 12:21 https://doi.org/10.1186/s13613-022-00982-1

In the original publication of the article [1], the legends of *x*- and *y*-axis of panel a in Fig. 1 was inadvertently omitted. Figure 1 should have appeared as shown in this correction (Fig. 1).

**Fig. 1:**
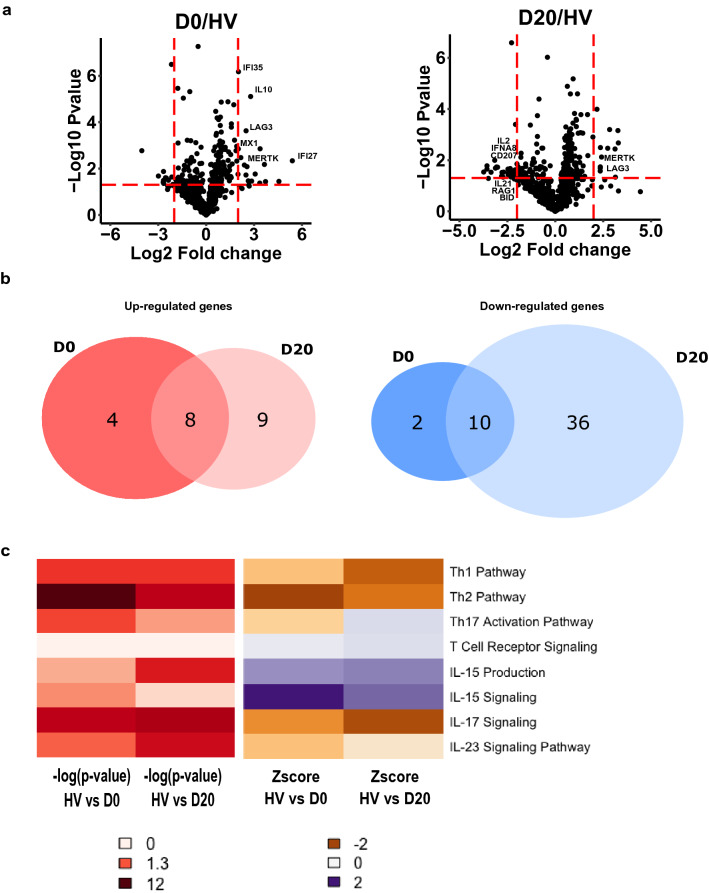
Transcriptomic profile of mononuclear cells in severe COVID-19 patients. RNA extracted from PBMCs of COVID-19 patients at day 0 and day 20 (*n* = 10) and healthy volunteers (HV, *n* = 10) was analyzed through NanoString technology. **a** Volcano plots of differentially expressed genes between patients sampled at D0 or at D20 and HV are showed. Limits of significance are illustrated by red dotted lines (i.e. Log2 Fold Change = −2 or + 2 and −Log 10 *P* value = 1.3). Selected genes are mentioned. **b** Venn diagrams of significantly up-regulated (left diagram, *n* = 21) or down-regulated (right diagram, *n* = 48) genes between patients and HV are showed. **c** Ingenuity Pathway Analysis was applied on the list of differentially expressed genes at D0 and D20. Heatmaps of Log 10 *P*-value (from white indicating the absence of significance to dark red indicating a strong significance) and *Z*-score (from orange indicating a down-regulation to purple indicating an up-regulation) for pathways related to T cell activation at D0 and D20 are presented

The original article has been corrected.
